# [μ-*N*,*N*,*N*′,*N*′-Tetra­kis(2-pyridylmeth­yl)­hexane-1,6-diamine]bis­[dichlorido­manganese(II)]

**DOI:** 10.1107/S1600536809003663

**Published:** 2009-02-04

**Authors:** In-Chul Hwang, Nam-Ho Kim, Kwang Ha

**Affiliations:** a Department of Chemistry, Pohang University of Science and Technology, Pohang 790-784, Republic of Korea; b School of Applied Chemical Engineering, The Research Institute of Catalysis, Chonnam National University, Gwangju 500-757, Republic of Korea

## Abstract

The asymmetric unit of the title compound, [Mn_2_Cl_4_(C_30_H_36_N_6_)], contains one-half of the formula unit; a centre of inversion is located at the mid-point of the mol­ecule. The two Mn^2+^ ions are bridged by the dual tri­dentate *N*,*N*,*N*′,*N*′-tetra­kis(2-pyridylmeth­yl)hexane-1,6-diamine ligand to form a dinuclear complex. Each Mn atom is five-coordinated in an approximately square-pyramidal geometry by three N atoms from the ligand and two Cl atoms. Inter­molecular π–π inter­actions between adjacent pyridine rings with a centroid–centroid distance of 3.576 (2) Å are reported.

## Related literature

For structural details of some related complexes, see: Hwang & Ha (2007 ▶); Song *et al.* (2008 ▶).
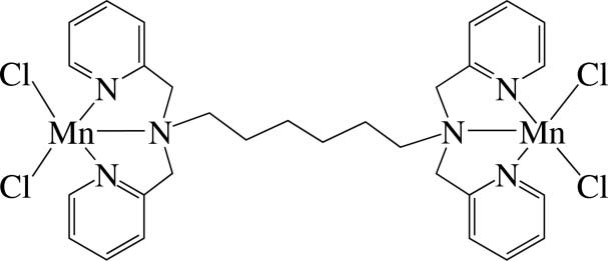


## Experimental

### 

#### Crystal data

[Mn_2_Cl_4_(C_30_H_36_N_6_)]
*M* *_r_* = 732.33
Triclinic, 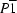

*a* = 7.7149 (13) Å
*b* = 8.4660 (14) Å
*c* = 14.263 (2) Å
α = 83.309 (3)°
β = 88.329 (3)°
γ = 66.666 (3)°
*V* = 849.4 (2) Å^3^
*Z* = 1
Mo *K*α radiation
μ = 1.09 mm^−1^
*T* = 293 (2) K
0.35 × 0.18 × 0.06 mm

#### Data collection

Bruker SMART 1000 CCD diffractometer
Absorption correction: multi-scan (*SADABS*; Bruker, 2000 ▶) *T*_min_ = 0.707, *T*_max_ = 0.937
4845 measured reflections
3368 independent reflections
2705 reflections with *I* > 2σ(*I*)
*R*_int_ = 0.013

#### Refinement

*R*[*F* ^2^ > 2σ(*F* ^2^)] = 0.043
*wR*(*F* ^2^) = 0.118
*S* = 1.07
3368 reflections
190 parameters
H-atom parameters constrained
Δρ_max_ = 0.42 e Å^−3^
Δρ_min_ = −0.29 e Å^−3^

### 

Data collection: *SMART* (Bruker, 2000 ▶); cell refinement: *SAINT* (Bruker, 2000 ▶); data reduction: *SAINT*; program(s) used to solve structure: *SHELXS97* (Sheldrick, 2008 ▶); program(s) used to refine structure: *SHELXL97* (Sheldrick, 2008 ▶); molecular graphics: *PLATON* (Spek, 2003 ▶); software used to prepare material for publication: *SHELXL97*.

## Supplementary Material

Crystal structure: contains datablocks global, I. DOI: 10.1107/S1600536809003663/cs2107sup1.cif

Structure factors: contains datablocks I. DOI: 10.1107/S1600536809003663/cs2107Isup2.hkl

Additional supplementary materials:  crystallographic information; 3D view; checkCIF report

## Figures and Tables

**Fig. 1.**
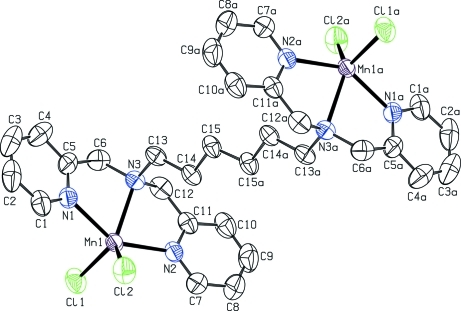
The structure of the title compound, with displacement ellipsoids drawn at the 50% probability level for non-H atoms [Symmetry code: (a) 1 - x, 1 - y, -z]. H atoms have been omitted for clarity.

**Fig. 2.**
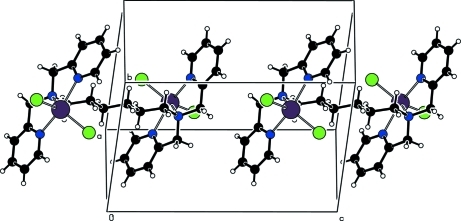
View of a packing detail of the title compound.

## supplementary crystallographic information

### Comment

The asymmetric unit of the title compound, [Mn_2_Cl_4_(C_30_H_36_N_6_)], contains one half of the formula unit; a centre of inversion is located in the midpoint of the compound (Figs. 1 and 2). In the complex, the two Mn^2+^ ions are bridged by the hexadentate ligand *N*,*N*,*N*',*N*'-tetrakis(2-pyridylmethyl)hexane-1,6-diamine (tphn) to form a centrosymmetric dinuclear complex. Mn atoms are five-coordinated in an approximately square pyramidal geometry by three N atoms from the tphn ligand and two Cl atoms. The Mn—*N*(amine) bond length (2.339 (2) Å) is slightly longer than the Mn—N(pyridyl) bond lengths (2.246 (3) and 2.232 (2) Å). The complex displays intermolecular π-π interactions between adjacent pyridine rings. The shortest distance between *Cg*1 (the centroid of six-membered ring N1—C5) and *Cg*1^i^ (symmetry code i: 2 - *x*, 1 - *y*, 1 - *z*) is 3.576 (2) Å.

### Experimental

To a solution of *N*,*N*,*N*',*N*'-tetrakis(2-pyridylmethyl)hexane-1,6-diamine (0.50 g, 1.04 mmol) in EtOH (15 ml) was added MnCl_2_.4H_2_O (0.21 g, 1.06 mmol) and stirred for 1 h at room temparature. The volume of the solvent was reduced to 3 ml and ether (20 ml) was added. The so formed precipitate was separated by filtration and washed with EtOH/ether and dried under vacuum, to give a pale yellow powder (0.36 g). Crystals suitable for X-ray analysis were obtained from the slow evaporation of a MeOH solution. MS (FAB): m/*z* 570, 572 (Mn(tphn)Cl^+^).

### Refinement

H atoms were positioned geometrically and allowed to ride on their respective parent atoms [C—H = 0.93 Å (aromatic) or 0.97 Å (CH_2_) and *U*_iso_(H) = 1.2*U*_eq_(C)].

### Crystal data

**Table:**

[Mn_2_Cl_4_(C_30_H_36_N_6_)]	*Z* = 1
*M**_r_* = 732.33	*F*(000) = 376
Triclinic, *P*1	*D*_x_ = 1.432 Mg m^−^^3^
Hall symbol: -P 1	Mo *K*α radiation, λ = 0.71073 Å
*a* = 7.7149 (13) Å	Cell parameters from 886 reflections
*b* = 8.4660 (14) Å	θ = 2.6–26.3°
*c* = 14.263 (2) Å	µ = 1.09 mm^−^^1^
α = 83.309 (3)°	*T* = 293 K
β = 88.329 (3)°	Plate, colourless
γ = 66.666 (3)°	0.35 × 0.18 × 0.06 mm
*V* = 849.4 (2) Å^3^	

### Data collection

**Table:**

Bruker SMART 1000 CCD diffractometer	3368 independent reflections
Radiation source: fine-focus sealed tube	2705 reflections with *I* > 2σ(*I*)
graphite	*R*_int_ = 0.013
φ and ω scans	θ_max_ = 26.4°, θ_min_ = 1.4°
Absorption correction: multi-scan (*SADABS*; Bruker, 2000)	*h* = −8→9
*T*_min_ = 0.707, *T*_max_ = 0.937	*k* = −10→10
4845 measured reflections	*l* = −17→15

### Refinement

**Table:**

Refinement on *F*^2^	Primary atom site location: structure-invariant direct methods
Least-squares matrix: full	Secondary atom site location: difference Fourier map
*R*[*F*^2^ > 2σ(*F*^2^)] = 0.043	Hydrogen site location: inferred from neighbouring sites
*wR*(*F*^2^) = 0.118	H-atom parameters constrained
*S* = 1.07	*w* = 1/[σ^2^(*F*_o_^2^) + (0.0564*P*)^2^ + 0.3638*P*] where *P* = (*F*_o_^2^ + 2*F*_c_^2^)/3
3368 reflections	(Δ/σ)_max_ = 0.001
190 parameters	Δρ_max_ = 0.42 e Å^−^^3^
0 restraints	Δρ_min_ = −0.29 e Å^−^^3^

### Fractional atomic coordinates and isotropic or equivalent isotropic displacement parameters (Å^2^)

**Table:**

	*x*	*y*	*z*	*U*_iso_*/*U*_eq_	
Mn1	1.04002 (6)	0.18359 (5)	0.26548 (3)	0.04078 (16)	
Cl1	1.23775 (14)	−0.01680 (11)	0.38692 (7)	0.0661 (3)	
Cl2	1.19512 (14)	0.26409 (14)	0.13691 (7)	0.0677 (3)	
N1	0.9580 (4)	0.4267 (3)	0.33551 (19)	0.0477 (6)	
N2	0.9371 (3)	0.0059 (3)	0.20318 (19)	0.0456 (6)	
N3	0.7153 (3)	0.2854 (3)	0.29208 (17)	0.0410 (6)	
C1	1.0607 (6)	0.5221 (5)	0.3353 (3)	0.0633 (10)	
H1	1.1711	0.4909	0.3008	0.076*	
C2	1.0105 (8)	0.6646 (5)	0.3840 (3)	0.0790 (14)	
H2	1.0868	0.7264	0.3839	0.095*	
C3	0.8461 (8)	0.7127 (5)	0.4324 (3)	0.0820 (15)	
H3	0.8101	0.8069	0.4670	0.098*	
C4	0.7338 (7)	0.6216 (5)	0.4298 (2)	0.0715 (12)	
H4	0.6178	0.6571	0.4595	0.086*	
C5	0.7963 (5)	0.4759 (4)	0.3822 (2)	0.0504 (8)	
C6	0.6920 (5)	0.3593 (4)	0.3819 (2)	0.0532 (8)	
H6A	0.7393	0.2665	0.4332	0.064*	
H6B	0.5589	0.4247	0.3919	0.064*	
C7	1.0319 (5)	−0.0980 (5)	0.1386 (3)	0.0601 (9)	
H7	1.1506	−0.1024	0.1210	0.072*	
C8	0.9591 (6)	−0.1979 (5)	0.0977 (3)	0.0752 (11)	
H8	1.0272	−0.2683	0.0527	0.090*	
C9	0.7868 (6)	−0.1926 (5)	0.1237 (3)	0.0767 (12)	
H9	0.7360	−0.2601	0.0967	0.092*	
C10	0.6872 (5)	−0.0877 (5)	0.1898 (3)	0.0634 (10)	
H10	0.5691	−0.0834	0.2086	0.076*	
C11	0.7668 (4)	0.0118 (4)	0.2281 (2)	0.0464 (7)	
C12	0.6656 (5)	0.1324 (4)	0.2993 (2)	0.0527 (8)	
H12A	0.5305	0.1708	0.2896	0.063*	
H12B	0.6977	0.0708	0.3622	0.063*	
C13	0.5988 (4)	0.4236 (4)	0.2181 (2)	0.0469 (7)	
H13A	0.6265	0.5251	0.2209	0.056*	
H13B	0.4667	0.4551	0.2332	0.056*	
C14	0.6265 (4)	0.3777 (4)	0.1184 (2)	0.0465 (7)	
H14A	0.6050	0.2731	0.1151	0.056*	
H14B	0.7557	0.3547	0.1005	0.056*	
C15	0.4928 (5)	0.5226 (4)	0.0496 (2)	0.0497 (8)	
H15A	0.3642	0.5508	0.0705	0.060*	
H15B	0.5200	0.6249	0.0505	0.060*	

### Atomic displacement parameters (Å^2^)

**Table:**

	*U*^11^	*U*^22^	*U*^33^	*U*^12^	*U*^13^	*U*^23^
Mn1	0.0360 (3)	0.0418 (3)	0.0450 (3)	−0.01554 (19)	−0.00546 (18)	−0.00439 (19)
Cl1	0.0736 (6)	0.0526 (5)	0.0663 (6)	−0.0198 (4)	−0.0295 (5)	0.0050 (4)
Cl2	0.0703 (6)	0.0923 (7)	0.0568 (6)	−0.0500 (5)	0.0078 (4)	−0.0076 (5)
N1	0.0516 (15)	0.0433 (14)	0.0476 (15)	−0.0178 (12)	−0.0088 (12)	−0.0046 (12)
N2	0.0448 (14)	0.0425 (14)	0.0498 (15)	−0.0177 (11)	−0.0097 (11)	−0.0021 (12)
N3	0.0406 (13)	0.0416 (13)	0.0382 (14)	−0.0157 (11)	−0.0018 (10)	0.0035 (10)
C1	0.070 (2)	0.055 (2)	0.070 (2)	−0.0309 (18)	−0.0226 (19)	0.0017 (18)
C2	0.126 (4)	0.050 (2)	0.068 (3)	−0.043 (2)	−0.047 (3)	0.012 (2)
C3	0.151 (5)	0.0364 (18)	0.046 (2)	−0.024 (2)	−0.030 (3)	0.0022 (16)
C4	0.106 (3)	0.0458 (19)	0.0382 (19)	−0.005 (2)	−0.0046 (19)	−0.0018 (15)
C5	0.068 (2)	0.0416 (16)	0.0297 (16)	−0.0101 (15)	−0.0117 (14)	0.0024 (13)
C6	0.0564 (19)	0.0550 (19)	0.0384 (18)	−0.0143 (16)	0.0050 (14)	0.0037 (14)
C7	0.055 (2)	0.059 (2)	0.067 (2)	−0.0202 (17)	−0.0004 (17)	−0.0171 (18)
C8	0.084 (3)	0.068 (2)	0.080 (3)	−0.032 (2)	−0.005 (2)	−0.027 (2)
C9	0.091 (3)	0.068 (2)	0.085 (3)	−0.044 (2)	−0.022 (2)	−0.013 (2)
C10	0.061 (2)	0.059 (2)	0.079 (3)	−0.0354 (18)	−0.0168 (19)	0.0048 (19)
C11	0.0487 (17)	0.0397 (15)	0.0500 (18)	−0.0200 (14)	−0.0132 (14)	0.0106 (14)
C12	0.0511 (18)	0.0532 (18)	0.056 (2)	−0.0268 (15)	−0.0002 (15)	0.0078 (16)
C13	0.0403 (16)	0.0483 (17)	0.0444 (18)	−0.0124 (14)	−0.0087 (13)	0.0080 (14)
C14	0.0429 (16)	0.0492 (17)	0.0429 (17)	−0.0159 (14)	−0.0110 (13)	0.0070 (14)
C15	0.0505 (18)	0.0470 (17)	0.0500 (18)	−0.0200 (14)	−0.0175 (14)	0.0072 (14)

### Geometric parameters (Å, °)

**Table:**

Mn1—N2	2.232 (2)	C6—H6B	0.9700
Mn1—N1	2.246 (3)	C7—C8	1.372 (5)
Mn1—N3	2.339 (2)	C7—H7	0.9300
Mn1—Cl2	2.3422 (10)	C8—C9	1.355 (6)
Mn1—Cl1	2.3716 (9)	C8—H8	0.9300
N1—C5	1.334 (4)	C9—C10	1.372 (6)
N1—C1	1.337 (4)	C9—H9	0.9300
N2—C11	1.334 (4)	C10—C11	1.386 (5)
N2—C7	1.340 (4)	C10—H10	0.9300
N3—C6	1.465 (4)	C11—C12	1.499 (5)
N3—C12	1.482 (4)	C12—H12A	0.9700
N3—C13	1.492 (4)	C12—H12B	0.9700
C1—C2	1.377 (6)	C13—C14	1.504 (4)
C1—H1	0.9300	C13—H13A	0.9700
C2—C3	1.362 (7)	C13—H13B	0.9700
C2—H2	0.9300	C14—C15	1.519 (4)
C3—C4	1.372 (6)	C14—H14A	0.9700
C3—H3	0.9300	C14—H14B	0.9700
C4—C5	1.386 (5)	C15—C15^i^	1.499 (6)
C4—H4	0.9300	C15—H15A	0.9700
C5—C6	1.501 (5)	C15—H15B	0.9700
C6—H6A	0.9700		
			
N2—Mn1—N1	145.81 (10)	C5—C6—H6B	109.4
N2—Mn1—N3	73.58 (9)	H6A—C6—H6B	108.0
N1—Mn1—N3	72.37 (9)	N2—C7—C8	122.0 (4)
N2—Mn1—Cl2	101.15 (8)	N2—C7—H7	119.0
N1—Mn1—Cl2	97.35 (8)	C8—C7—H7	119.0
N3—Mn1—Cl2	128.09 (6)	C9—C8—C7	119.1 (4)
N2—Mn1—Cl1	98.49 (7)	C9—C8—H8	120.4
N1—Mn1—Cl1	98.94 (7)	C7—C8—H8	120.4
N3—Mn1—Cl1	116.01 (7)	C8—C9—C10	120.0 (4)
Cl2—Mn1—Cl1	115.84 (4)	C8—C9—H9	120.0
C5—N1—C1	118.3 (3)	C10—C9—H9	120.0
C5—N1—Mn1	116.8 (2)	C9—C10—C11	118.4 (4)
C1—N1—Mn1	124.9 (3)	C9—C10—H10	120.8
C11—N2—C7	118.7 (3)	C11—C10—H10	120.8
C11—N2—Mn1	117.6 (2)	N2—C11—C10	121.8 (3)
C7—N2—Mn1	123.6 (2)	N2—C11—C12	116.5 (3)
C6—N3—C12	112.0 (2)	C10—C11—C12	121.7 (3)
C6—N3—C13	108.5 (2)	N3—C12—C11	111.9 (3)
C12—N3—C13	111.5 (2)	N3—C12—H12A	109.2
C6—N3—Mn1	104.43 (18)	C11—C12—H12A	109.2
C12—N3—Mn1	106.39 (18)	N3—C12—H12B	109.2
C13—N3—Mn1	113.75 (18)	C11—C12—H12B	109.2
N1—C1—C2	122.9 (4)	H12A—C12—H12B	107.9
N1—C1—H1	118.5	N3—C13—C14	115.8 (3)
C2—C1—H1	118.5	N3—C13—H13A	108.3
C3—C2—C1	118.3 (4)	C14—C13—H13A	108.3
C3—C2—H2	120.8	N3—C13—H13B	108.3
C1—C2—H2	120.8	C14—C13—H13B	108.3
C2—C3—C4	119.8 (4)	H13A—C13—H13B	107.4
C2—C3—H3	120.1	C13—C14—C15	111.5 (3)
C4—C3—H3	120.1	C13—C14—H14A	109.3
C3—C4—C5	118.8 (4)	C15—C14—H14A	109.3
C3—C4—H4	120.6	C13—C14—H14B	109.3
C5—C4—H4	120.6	C15—C14—H14B	109.3
N1—C5—C4	121.8 (4)	H14A—C14—H14B	108.0
N1—C5—C6	115.2 (3)	C15^i^—C15—C14	113.2 (3)
C4—C5—C6	123.0 (4)	C15^i^—C15—H15A	108.9
N3—C6—C5	111.0 (3)	C14—C15—H15A	108.9
N3—C6—H6A	109.4	C15^i^—C15—H15B	108.9
C5—C6—H6A	109.4	C14—C15—H15B	108.9
N3—C6—H6B	109.4	H15A—C15—H15B	107.8
			
N2—Mn1—N1—C5	25.2 (3)	C1—N1—C5—C4	0.0 (4)
N3—Mn1—N1—C5	19.9 (2)	Mn1—N1—C5—C4	178.6 (2)
Cl2—Mn1—N1—C5	147.7 (2)	C1—N1—C5—C6	−178.1 (3)
Cl1—Mn1—N1—C5	−94.6 (2)	Mn1—N1—C5—C6	0.5 (3)
N2—Mn1—N1—C1	−156.3 (2)	C3—C4—C5—N1	−3.1 (5)
N3—Mn1—N1—C1	−161.6 (3)	C3—C4—C5—C6	174.9 (3)
Cl2—Mn1—N1—C1	−33.9 (3)	C12—N3—C6—C5	162.7 (3)
Cl1—Mn1—N1—C1	83.9 (3)	C13—N3—C6—C5	−73.7 (3)
N1—Mn1—N2—C11	−20.5 (3)	Mn1—N3—C6—C5	47.9 (3)
N3—Mn1—N2—C11	−15.3 (2)	N1—C5—C6—N3	−34.7 (4)
Cl2—Mn1—N2—C11	−142.0 (2)	C4—C5—C6—N3	147.2 (3)
Cl1—Mn1—N2—C11	99.5 (2)	C11—N2—C7—C8	−0.1 (5)
N1—Mn1—N2—C7	155.5 (2)	Mn1—N2—C7—C8	−176.0 (3)
N3—Mn1—N2—C7	160.7 (3)	N2—C7—C8—C9	−0.5 (6)
Cl2—Mn1—N2—C7	34.0 (3)	C7—C8—C9—C10	0.3 (7)
Cl1—Mn1—N2—C7	−84.5 (3)	C8—C9—C10—C11	0.4 (6)
N2—Mn1—N3—C6	147.77 (19)	C7—N2—C11—C10	0.8 (5)
N1—Mn1—N3—C6	−35.30 (18)	Mn1—N2—C11—C10	177.0 (2)
Cl2—Mn1—N3—C6	−120.79 (17)	C7—N2—C11—C12	−178.9 (3)
Cl1—Mn1—N3—C6	56.21 (19)	Mn1—N2—C11—C12	−2.7 (3)
N2—Mn1—N3—C12	29.15 (19)	C9—C10—C11—N2	−0.9 (5)
N1—Mn1—N3—C12	−153.9 (2)	C9—C10—C11—C12	178.8 (3)
Cl2—Mn1—N3—C12	120.59 (18)	C6—N3—C12—C11	−154.0 (3)
Cl1—Mn1—N3—C12	−62.4 (2)	C13—N3—C12—C11	84.1 (3)
N2—Mn1—N3—C13	−94.1 (2)	Mn1—N3—C12—C11	−40.4 (3)
N1—Mn1—N3—C13	82.9 (2)	N2—C11—C12—N3	30.8 (4)
Cl2—Mn1—N3—C13	−2.6 (2)	C10—C11—C12—N3	−148.9 (3)
Cl1—Mn1—N3—C13	174.37 (18)	C6—N3—C13—C14	171.8 (3)
C5—N1—C1—C2	2.5 (5)	C12—N3—C13—C14	−64.3 (3)
Mn1—N1—C1—C2	−176.0 (3)	Mn1—N3—C13—C14	56.0 (3)
N1—C1—C2—C3	−1.8 (5)	N3—C13—C14—C15	176.4 (2)
C1—C2—C3—C4	−1.4 (6)	C13—C14—C15—C15^i^	−176.3 (3)
C2—C3—C4—C5	3.7 (5)		

Symmetry codes: (i) −*x*+1, −*y*+1, −*z*.
